# Telmisartan, an AT1 receptor blocker and a PPAR gamma activator, alleviates liver fibrosis induced experimentally by *Schistosoma mansoni* infection

**DOI:** 10.1186/1756-3305-6-199

**Published:** 2013-07-05

**Authors:** Yasmeen M Attia, Essam F Elalkamy, Olfat A Hammam, Soheir S Mahmoud, Aiman S El-Khatib

**Affiliations:** 1 Department of Pharmacology and Biochemistry, Faculty of Pharmacy, The British University in Egypt, Suez Desert Road, P.O. Box 43, El Sherouk City, Cairo 11837, Egypt; 2 Department of Pharmacology, Faculty of Medicine, Cairo University, Al-Saray St., El Manial, Cairo 11956, Egypt; 3 Department of Pathology, Theodor Bilharz Research Institute, Warrak El-Hadar, Imbaba, P.O. Box 30, Giza 12411, Egypt; 4 Department of Parasitology, Theodor Bilharz Research Institute, Warrak El-Hadar, Imbaba, P.O. Box 30, Giza 12411, Egypt; 5 Department of Pharmacology and Toxicology, Faculty of Pharmacy, Cairo University, Kasr El-Aini St., Cairo 11562, Egypt

**Keywords:** Hepatic fibrosis, *Schistosoma mansoni*, Telmisartan, MMP-2, TIMP-2, TGF-β1

## Abstract

**Background:**

Hepatic schistosomiasis is considered to be one of the most prevalent forms of chronic liver disease in the world due to its complication of liver fibrosis. The demonstration of the pro-fibrogenic role of angiotensin (Ang) II in chronic liver disease brought up the idea that anti-Ang II agents may be effective in improving hepatic fibrosis by either blocking Ang II type 1 (AT1) receptors or inhibiting the angiotensin converting enzyme. Peroxisome proliferator-activated receptors gamma (PPARγ) activation has been also shown to inhibit hepatic stellate cell activation and progression of fibrosis. The present study has aimed at testing the anti-fibrogenic effects of telmisartan; an AT1 receptor blocker and a PPARγ partial agonist, alone or combined with praziquantel (PZQ) on *Schistosoma mansoni*-induced liver fibrosis in mice.

**Methods:**

To achieve the aim of the study, two sets of experiments were performed in which telmisartan was initiated at the 5^th^ (set 1) and the 10^th^ (set 2) weeks post infection to assess drug efficacy in both acute and chronic stages of liver fibrosis, respectively. *Schistosoma mansoni*-infected mice were randomly divided into the following four groups: infected-control (I), telmisartan-treated (II), PZQ-treated (III), and telmisartan+PZQ-treated (IV). In addition, a normal non-infected group was used for comparison. Parasitological (hepatomesenteric worm load and oogram pattern), histopathological, morphometric, immunohistochemical (hepatic expressions of matrix metalloproteinase-2; MMP-2 and tissue inhibitor of metalloproteinase-2; TIMP-2), and biochemical (serum transforming growth factor beta 1; TGF-β1 and liver function tests) studies were performed.

**Results:**

Telmisartan failed to improve the parasitological parameters, while it significantly (P<0.05) decreased the mean granuloma diameter, area of fibrosis, and serum TGF-β1. Additionally, telmisartan increased MMP-2 and decreased TIMP-2 hepatic expression. Combined treatment failed to show any additive properties, yet it did not affect the anti-schistosomal activity of PZQ.

**Conclusions:**

These results suggest potential anti-fibrotic effects of telmisartan, an AT1 receptor blocker and a PPARγ partial agonist, in acute and chronic stages of *Schistosoma mansoni*–induced liver fibrosis in mice.

## Background

In recent years, there has been an increased recognition of the importance of Neglected Tropical Diseases (NTDs) as impediments to development. The World Health Organization (WHO) has defined seventeen of these conditions as core NTDs [1]. One of these NTDs is schistosomiasis which is a major parasitic disease, caused by blood flukes (trematode worms) of the genus *Schistosoma* (*S*.), affecting millions of people worldwide. It is endemic in about 76 countries and territories, though it is estimated that 85% of the infected people are on the African continent [2]. Chemotherapy with praziquantel (PZQ) is the mainstay of schistosomiasis control. Yet there are recent concerns about tolerance or resistance to PZQ, and hence monitoring its efficacy in different settings is required [3].

Inflammatory hepatic schistosomiasis, which is the main cause of schistosomal hepatomegaly in children and adolescents, is an early reaction to the ova trapped in the perisinusoidal periportal spaces of the liver [4]. Peri-portal fibrosis is considered a serious consequence of *S*. *mansoni* infection that involves remodeling of extracellular matrix (ECM) and excessive deposition of collagen, primarily by hepatic stellate cells (HSCs), along the branches of the portal tract [5].

Over the last few years, HSCs have been commonly recognized as the principal cell type responsible for ECM protein formation during hepatic fibrogenesis, and the cytokine transforming growth factor beta 1 (TGF-β1) represents one key factor stimulating collagen and ECM production in these cells [6]. The cytokine TGF-β1 promotes wound healing and repair. Under pathological conditions, TGF-β1 orchestrates a cross talk between parenchymal, inflammatory, and collagen-expressing cells and plays a key role in stimulating fibrosis. Additionally, overexpression of TGF-β1 in transgenic mice results in fibrosis of multiple organs, suggesting that TGF-β1 is a major pro-fibrogenic cytokine [7].

Complete recovery from liver fibrosis involves remodelling and breakdown of multiple ECM components, with degradation of the predominant component, collagen I, being particularly important for recovery of normal liver histology. An enlarging family of matrix metalloproteinases (MMPs) was identified, which are calcium-dependent enzymes that specifically degrade collagenous and non-collagenous substrates [8]. Loebermann *et al*. [9] reported that the expression of MMP-2 correlates with both the onset and progression of fibrosis. However, there is increasing evidence that collagenase inhibition may arise from increased expression of endogenous MMP inhibitors, tissue inhibitor of metalloproteinases (TIMPs) in fibrotic liver. Expressions of both TIMP-1 and -2 were found to be elevated in human and rat models of liver fibrosis [10]. The resulting increase in TIMP:MMP ratio in liver may promote fibrosis by protecting deposited ECM from degradation by MMPs [11].

The renin-angiotensin system (RAS) has been shown to play a pivotal role in hepatic fibrogenesis [12]. A common observation in most of the previous studies is that liver injury is associated with an up-regulation of RAS components including angiotensinogen, renin, angiotensin converting enzyme, angiotensin (Ang) II, and Ang II type-1 (AT1) receptors [13]. The balance between ECM deposition and degradation, which depends on the relative activity of MMPs and their inhibitors, TIMPs, was also found to be affected by hepatic RAS. Following injury, AT1 receptor expression on activated HSCs is increased and these cells demonstrate increased responsiveness to Ang II compared to quiescent HSCs [14]. Angiotensin II has been shown to provoke pro-fibrotic and pro-inflammatory effects on HSCs including ECM production and fibrotic markers such as α-smooth muscle actin and collagen, and the expression of inflammatory cytokines and growth factors such as TGF-β1 [12].

Peroxisome proliferator activated receptor gamma (PPARγ) is a ligand-activated nuclear transcription factor that belongs to the nuclear hormone receptor superfamily. Hazra *et al*. [15] reported that PPARγ depletion is associated with HSC activation, whereas increasing PPARγ expression induces HSC quiescence and has been found to inhibit activation markers such as α-smooth muscle actin and collagen. Moreover, PPARγ receptors were found to have anti-proliferative and anti-fibrotic effects on activated HSCs as well as inducing HSCs apoptosis through a mechanism involving an extrinsic apoptosis pathway [16].

Previous studies have shown that telmisartan (TELM) is distinguished from other members of AT1 receptor blockers by its partial agonistic activity on PPARγ receptors, which was previously reported to have anti-inflammatory and anti-oxidant properties [17,18]. Accordingly, the present study has been focused on investigating the potential effect of a drug with combined effects such as TELM, an AT1 receptor blocker and a PPARγ activator, either alone or combined with PZQ, on *S*. *mansoni*-induced liver fibrosis in mice.

## Methods

### Animals

Swiss male albino mice of CD-1 strain, weighing 18–20 g each, were provided by the Schistosome Biology Supply Center (SBSC), Theodor Bilharz Research Institute (TBRI), Giza, Egypt. They were fed on a standard diet with free access to water at the animal house of the SBSC of TBRI. The animals were kept under standard conditions of temperature (25±0.5°C), relative humidity (55±1%) and light cycle (12 h light and 12 h dark). All experiments were conducted in accordance with the international ethical guidelines and were approved by the Ethics Committee of the TBRI.

### Infection

An Egyptian strain of *Schistosoma mansoni* cercariae was provided by the SBSC of TBRI. Cercariae were shed from laboratory bred infected snails namely, *Biomphalaria alexandrina*, 25–30 days after exposure to miracidia, according to the method described by Pellegrino *et al*. [19]. Infection was carried out by subcutaneous injection of mice with 60±10 *S*. *mansoni* cercariae suspended in 0.2 ml solution [20].

### Drugs and doses

Praziquantel (E.I.P.I.Co. Pharmaceuticals, Cairo, Egypt) was prepared as suspension in Cremophor-El and given orally seven weeks post infection (WPI) at a dose of 500 mg/kg/day for two consecutive days [21]. Telmisartan (Boehringer, Ingelheim, Germany) was given orally at a dose of 10 mg/kg/day [22] for five weeks. In accordance with the experimental design, it was started at either five or ten WPI.

### Experimental design

Two sets of experiments were performed. In the first set, TELM treatment was initiated five WPI and in the second set, it was started ten WPI. In each set, infected mice were randomly allocated to the following four groups, each consisting of ten mice:

### Group I (infected control)

This group received only the drug vehicle, p.o.

### Group II (TELM-treated)

This group received TELM for five weeks.

### Group III (PZQ-treated)

This group received PZQ, seven WPI for two consecutive days.

### Group IV (TELM+PZQ-treated)

This group received both TELM and PZQ as indicated in groups II and III.

For comparison, a universal group (normal non-infected), consisted of 20 mice (ten for each set) was used.

All animal groups belonging to either set of experiments, were sacrificed by decapitation at the end of TELM treatment for carrying out the selected parasitological, histopathological, morphometric, immunohistochemical, and biochemical studies.

### Parasitological studies

At the 10^th^ and 15^th^ WPI, all animals were sacrificed and perfused using a Masterflex pump (Cole-Parmer Instrument Company, USA). Worms recovered from the hepatic and mesenteric compartments were collected and counted. The anti-schistosomal effect of the drug was assessed parasitologically by assessing the *S*. *mansoni* hepatomesenteric worm load [23], and the oogram pattern to determine the percentage of the different egg developmental stages in the small intestines of mice [19].

### Biochemical studies

Blood samples collected from sacrificed mice were allowed to stand for 30 min before centrifugation at 3000 rpm, for 15 min. Sera were then separated and stored at -80°C for further estimation of:

#### Alanine transaminase (ALT) and aspartate transaminase (AST) enzyme activities

Serum levels of ALT and AST were estimated using the available commercial kits (Roche Diagnostics, Germany).

#### TGF-β1

Serum TGF-β1 was detected using an enzyme-linked immunosorbent assay (ELISA) kit (R&D Systems, USA) in accordance with the manufacturer’s instructions.

### Histopathological studies

Livers were excised from sacrificed mice, immediately fixed in 10% formalin solution and embedded in paraffin. Histological sections were processed and stained with hematoxylin and eosin (H&E) to examine the histopathological changes and with Masson’s Trichome to measure the mean granuloma diameter (μm) using an ocular micrometer (Zeiss, Germany), according to the method described by von Lichtenberg [24].

### Morphometric Studies

Hepatic sections, 20 μm in thickness, were prepared and stained with sirius red for the quantitation of the collagen content using the computer-controlled Image Analysis System (Leica, USA). Image analysis was performed using the computer software program KS 200.

The sectional area of the red stained fibrous tissue of the examined specimen was measured in squared microns in five consecutive microscopic fields, at X125 magnification to yield the fibrotic area (μm^2^). Fibrotic area relative to the total area (%) was then calculated as described by Coutinho *et al*. [25]. For each group, the results of all fibrotic areas (μm^2^) and fibrotic areas (%) were then averaged.

### Immunohistochemical studies

Hepatic sections, 4 μm in thickness, were mounted on glass slides pre-treated with 3-amino propyl-triethoxy saline (TESPA). The standard avidin-biotin immunoperoxidase technique was used [26]. Hepatic paraffin sections were de-waxed in xylene and hydrated in descending grades of ethanol. The endogenous peroxidase activity was quenched by incubation in 100% methanol with 30% hydrogen peroxide for 30 min. Antigen retrieval was performed by placing the slides in a jar filled with 20 ml antigen retrieval solution (Dako, Denmark) and 180 ml distilled water in a water bath for 15 min of microwaves at 700 W. Sections were incubated overnight at 4°C in a humid chamber with primary antibodies against MMP-2 and TIMP-2 (Santa Cruz Biotechnology, Santa Cruz, CA., USA). The antibodies were diluted 1:100, 1:100, respectively, in phosphate buffered saline (PBS). After rinsing in PBS, the sections were incubated at room temperature for 30 min with biotinylated secondary anti-mouse antibody (DAKO, Denmark) and after a further wash in PBS, the slides were incubated with an avidin-biotin complex horseradish peroxidase solution (DAKO, Denmark) for another 30 min. Slides were then washed with PBS for 5 min. After 10 minutes incubation, the peroxidase reaction was developed using 0.01% hydrogen peroxide in 0.05% diaminobenzidine tetrahydrochloride (DAB). Sections were counterstained with Mayer's hematoxylin and dehydrated in ascending grades of ethanol prior to mounting. Liver sections, with the primary antibody replaced by PBS, served as negative controls, while colonic cancer sections served as MMP-2 and TIMP-2 positive controls. Immunostaining interpretation of either MMP-2 or TIMP-2 was carried out according to the method described by Sinicrope *et al*. [27]. Liver sections were examined using a light microscope (Zeiss, Germany). The number of positively stained cells with the highest expression recorded within ten successive fields (X400) was counted per section/animal in a semi-quantitative way for both markers and their mean was calculated. For each group, the mean percentages were then averaged. Zero percentage was given to unstained sections.

### Statistical analysis

All values are presented as means ± S.E.M. Mean data from each study group were compared by one-way analysis of variance (one-way ANOVA). Chi-square test was used for percent positivity. P values less than 0.05 were considered to be statistically significant.

## Results

### Parasitological studies

At the 10^th^ and 15^th^ WPI, treatment with TELM alone and combined with PZQ failed to cause any significant changes in either the hepatomesenteric worm load or the oogram pattern, as compared to the infected control and PZQ-treated groups, respectively. However, PZQ treatment caused significant reduction in the hepatomesentric worm load; by 91.13 and 94.99%, at the 10^th^ and 15^th^ WPI, respectively, as compared to the infected control groups. Furthermore, at the 10^th^ WPI, PZQ caused 100 and 90.68% reductions in both immature and mature egg counts, respectively, as well as a 13.22-fold increase in dead eggs, as compared to the infected control group. At the 15^th^ WPI, treatment with PZQ showed 100 and 97.27% reductions in immature and mature egg counts, respectively, in addition to a 5.07-fold increase in the dead egg count, compared to the infected control group (Table 1).

**Table 1:** **Effects of 5 weeks treatment with telmisartan (TELM) given either alone or combined with praziquantel (PZQ) on hepatomesenteric worm load and oogram pattern (% egg developmental stages) of mice infected with *Schistosoma mansoni* and sacrificed at the 10**^**th **^**(set 1) and 15**^**th **^**(set 2) weeks post infection**

		**Set 1**				**Set 2**			
		**Infected control**	**TELM-treated**	**PZQ-treated**	**TELM+PZQ-treated**	**Infected control**	**TELM-treated**	**PZQ-treated**	**TELM+PZQ-treated**
**Hepatomesenteric worm load**	**Male**	5.25±0.12	5.75±0.51	1.17±0.08*^@^	0.72±0.03*^@^	3.86±0.28	4.56±0.06	0.63±0.03* ^@^	1.10±0.09* ^@^
	**Female**	1.75±0.13	1.88±0.04	0.17±0.01*^@^	0.14±0.01*^@^	1.86±0.11	1.67±0.13	0.00±0.00*^@^	0.00±0.00*^@^
	**Couple**	4.00±0.41	3.25±0.37	0.00±0.00*^@^	0.00±0.00*^@^	3.72±0.08	3.56±0.18	0.00±0.00*^@^	0.00±0.00*^@^
	**Total**	15.00±1.08	14.25±0.88	1.33±0.12*^@^	0.86±0.03*^@^	12.57±0.65	12.56±0.41	0.63±0.03*^@^	1.10±0.08*^@^
**% Egg developmental stages**	**Immature**	48.00±2.00	52.13±0.74	0.00±0.00*^@^	0.00±0.00*^@^	40.57±0.61	38.67±1.29	0.00±0.00*^@^	0.00±0.00*^@^
	**Mature**	44.75±1.93	41.00±0.54	4.17±2.00*^@^	3.29±0.01*^@^	36.57±1.11	35.22±1.73	1.00±0.05*^@^	0.70±0.02*^@^
	**Dead**	7.25±0.02	6.88±0.48	95.83±2.01*^@^	96.72±0.72*^@^	19.58±1.56	20.33±0.67	99.13±0.44*^@^	98.60±0.65*^@^

Mice were infected with 60±10 cercariae/mouse, injected subcutaneously. Telmisartan (TELM; 10 mg/kg/day, p.o.) was given at the 5^th^ (set 1) and 10^th^ (set 2) weeks post infection for 5 weeks. Praziquantel (PZQ; 500 mg/kg/day, p.o.) was given at the 7^th^ week post infection for 2 consecutive days. Animals were sacrificed at the end of TELM treatment. Data are represented as mean ± S.E.M. (n = 8–10). Significant difference (P < 0.05) * versus infected control group and ^@^ versus TELM-treated group.

### Biochemical studies

**Figure 1:**
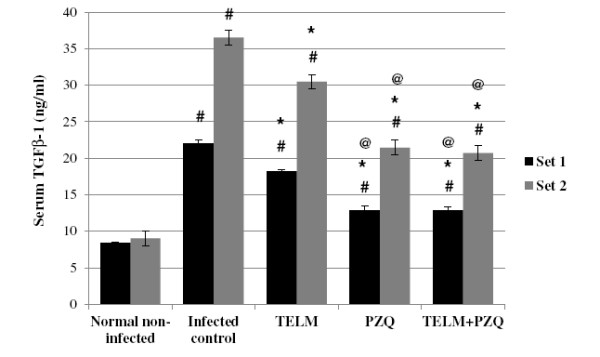
**Effect of telmisartan (TELM; 10 mg/kg/day, for 5 weeks), praziquantel (PZQ; 500 mg/kg/day, for 2 consecutive days), and their combination on the serum levels of transforming growth factor β1 (TGF-β1) in ng/ml of *Schistosma mansoni*-infected mice sacrificed at the 10**^**th **^**(set 1) and 15**^**th **^**(set 2) weeks post infection.** All values are mean ± S.E.M. Significantly different (P<0.05) ^#^ versus normal non-infected group, * versus infected control group and ^@^ versus TELM-treated group.

At the 10^th^ and 15^th^ WPI, the serum TGF-β1 measured in the infected control groups revealed 2.62- and 4.04-fold increases, respectively, compared to the normal non-infected groups. Moreover, the *S*. *mansoni* infection caused elevations in serum levels of ALT and AST by 1.6- and 2.33-fold at the 10^th^ WPI, and by 2.01- and 2.18- fold at the 15^th^ WPI, respectively, compared to the normal non-infected groups. TELM- and PZQ-treated groups showed significant reductions in serum TGF-β1 by 17.37% and 41.66% at the 10^th^ WPI and by 16.57% and 41.29% at the 15^th^ WPI, respectively, compared to their corresponding infected control groups. This showed that at the first and second sets, the reductions in the serum TGF-β1 levels observed with PZQ treatment were higher by 2.39 and 2.49 fold than that observed with TELM. Treatment with TELM combined with PZQ did not produce significant reductions in serum TGF-β1 levels in both sets, when compared to PZQ treatment alone (Figure 1).

**Figure 2:**
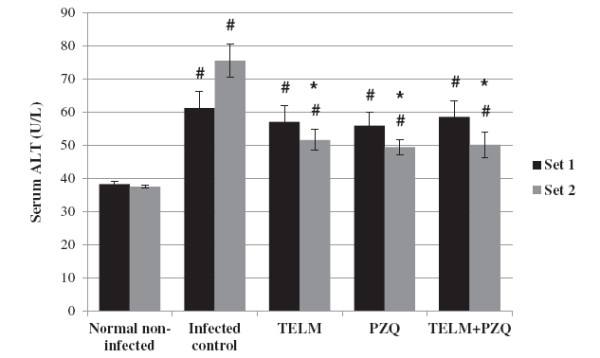
**Effect of temisartan (TELM; 10 mg/kg/day, for 5 weeks), praziquantel (PZQ; 500 mg/kg/day, for 2 consecutive days), and their combination on the serum levels of alanine transaminase (ALT) in U/L of *Schistosma mansoni*-infected mice sacrificed at the 10**^**th **^**(set 1) and 15**^**th **^**(set 2) weeks post infection.** All values are mean ± S.E.M. Significantly different (P<0.05) ^#^ versus normal non-infected group and * versus infected control group.

**Figure 3:**
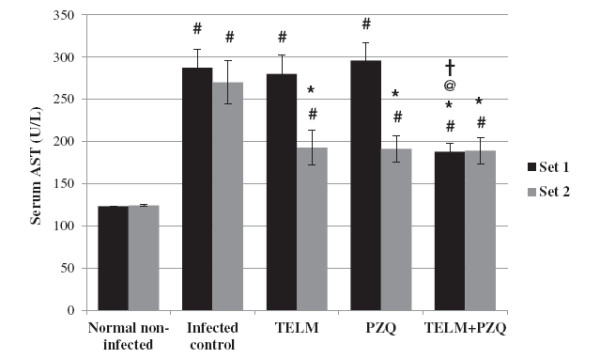
**Effect of telmisartan (TELM; 10 mg/kg/day, for 5 weeks), praziquantel (PZQ; 500 mg/kg/day, for 2 consecutive days), and their combination on the serum levels of aspartate transaminase (AST) in U/L of *Schistosma mansoni*-infected mice sacrificed at the 10**^**th **^**(set 1) and 15**^**th **^**(set 2) weeks post infection.** All values are mean ± S.E.M. Significantly different (P<0.05) ^#^ versus normal non-infected group, * versus infected control group, ^@^ versus TELM-treated group, and ^†^ versus PZQ-treated group.

At the 10^th^ WPI, TELM- and PZQ-treated groups did not show any significant change in serum ALT and AST levels in comparison with the infected control group. Moreover, combined treatment did not affect serum ALT levels significantly, while it caused serum AST levels to decrease by 34.52%, when compared to the infected control group. However, at the 15^th^ WPI, TELM- and PZQ-treated groups showed reductions in serum levels of ALT by 31.63 and 34.49% and in AST by 28.72 and 29.24%, respectively, compared to the infected control group. Moreover, treatment with PZQ combined with TELM decreased both ALT and AST serum levels by approximately 30%, compared to the infected control group of the second set. It did not show any significant differences, however, when compared to the reductions observed in the corresponding TELM- and PZQ-treated groups (Figures 2 and 3).

### Morphometric studies

As shown in Table 2, TELM- and PZQ-treated groups showed reductions in the estimated fibrotic areas by 32.78 and 70.53% at the 10^th^ WPI and by 43.06 and 54.75% at the 15^th^ WPI, respectively, compared to the corresponding infected control groups. At the 10^th^ WPI, the reduction in the area of fibrosis observed in the PZQ-treated group was 2.82-fold higher than that observed in the TELM-treated one. Combining TELM with PZQ did not show any significant change in the fibrotic area measured, when compared to PZQ-treated groups at both treatment periods.

**Table 2:** **Effects of 5 weeks treatment with telmisartan (TELM) given either alone or combined with praziquantel (PZQ) on the mean granuloma diameter (μm), immunohistochemical expression of matrix metalloproteinase-2 (MMP-2) and tissue inhibitor of metalloproteinase-2 (TIMP-2) expressed as % of positively stained cells, and fibrotic area in μm**^**2 **^**and % of fibrotic area relative to the total area examined of mice infected with *Schistosoma mansoni *and sacrificed at the 10**^**th **^**(set 1) and 15**^**th **^**(set 2) weeks post infection**

	**Set 1**				**Set 2**			
	**Infected control**	**TELM-treated**	**PZQ-treated**	**TELM+PZQ-treated**	**Infected control**	**TELM-treated**	**PZQ-treated**	**TELM+PZQ-** **treated**
Mean granuloma diameter (μm)	286.33±5.07	240.10±11.41*	228.17±6.87*	234.72±5.54*	236.72±8.04	171.3±5.09*	158.25±7.38*	165.30±5.01*
MMP-2 (%)	38.75±3.07	40.75±0.39	37.1±2.89	37.18±2.91	9.29±0.07	39.45±3.27*	10.38±0.20^@^	37.00±3.61*^†^
TIMP-2 (%)	24.75±1.70	5.75±0.96*	13.33±1.12*^@^	6.38±0.49*^@^	34.14±2.89	7.78±0.53*	32.88±3.05^@^	8.58±0.81*^†^
Fibrotic area (μm^2^)	20.10±1.23	13.51±0.48*	5.92±0.67*^@^	5.59±0.71*^@^	12.75±1.01	7.26±0.56*	5.77±0.58*	5.82±0.88*
Fibrotic area (%)	7.36±0.17	4.94±0.29*	2.25±0.26*^@^	2.14±0.27*^@^	4.84±0.38	2.76±0.21*	2.20±0.21*	2.22±0.32*

Mice were infected with 60±10 cercariae/mouse, injected subcutaneously. Telmisartan (TELM; 10 mg/kg/day, p.o.) was given at the 5^th^ (set 1) and 10^th^ (set 2) weeks post infection for 5 weeks. Praziquantel (PZQ; 500 mg/kg/day, p.o.) was given at the 7^th^ week post infection for 2 consecutive days. Animals were sacrificed at the end of TELM treatment. Data are represented as mean ± S.E.M (n = 8-10). Significant difference (P < 0.05) * versus infected control group, ^@^ versus TELM-treated group, and ^†^ versus PZQ-treated group.

### Histopathological studies

At the 10^th^ WPI, mice infected with *S*. *mansoni* showed large fibrocellular granulomas in the hepatic parenchyma with an outer zone of inflammatory cells mainly eosinophils, neutrophils, and lymphocytes and an inner zone of fibrous tissue encircling the living bilharzial ova. The mean granuloma diameter was found to be 286.33±5.07 μm. On the other hand, mice sacrificed at the 15^th^ WPI showed more granulomas deposited in the hepatic parenchyma with more dense fibrous tissue and a decrease in the inflammatory outer zone where the mean granuloma diameter was found to be 236.72±8.04 μm (Table 2).

Telmisartan treatment showed a regression of the granulomatous inflammatory reaction resulting in a decrease in the mean diameter of the fibrocellular granuloma at the 10^th^ and 15^th^ WPI by 16.15 and 27.64%, respectively, compared to the infected control groups (Table 2).

Treatment with PZQ showed fibrocellular granulomas formed of degenerated ova surrounded by giant cells, pigmented macrophages, lymphocytes, plasma cells, and fibrous tissue. Moreover, a decrease in the mean granuloma diameter was observed in the PZQ-treated groups at the 10^th^ and 15^th^ WPI by 20.31 and 33.15%, respectively, as compared to the infected control groups (Table 2).

Combining PZQ with TELM did not produce any significant change in the mean granuloma diameter compared to either TELM- or PZQ-treated groups at both treatment periods (Table 2).

### Immunohistochemical studies

**Figure 4:**
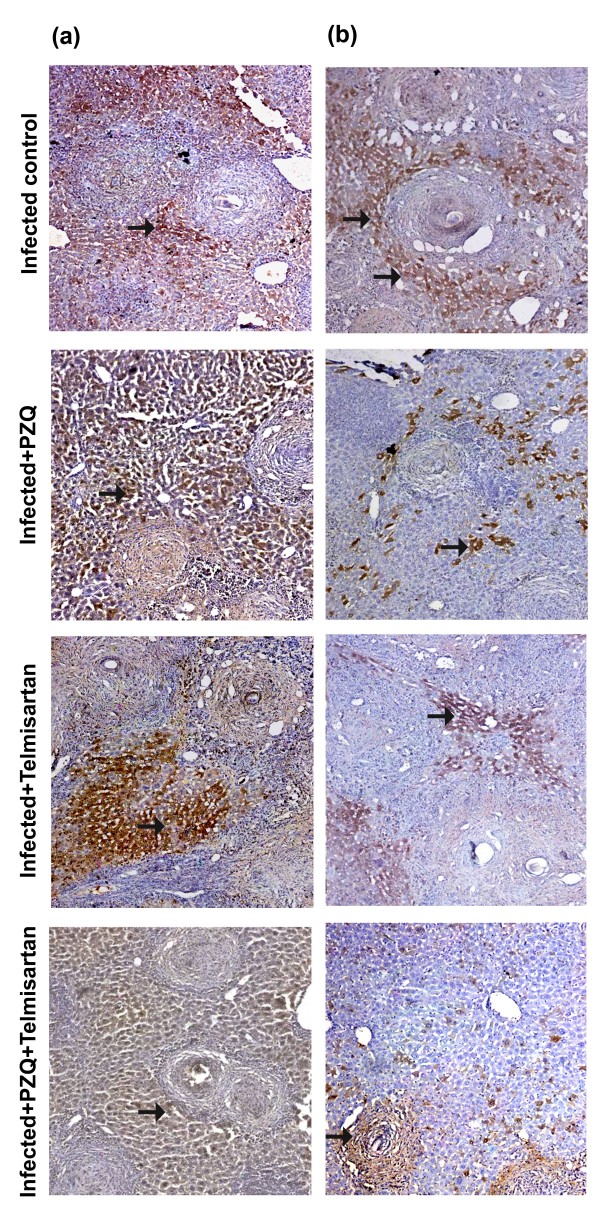
**Immunostain for matrix metalloproteinase-2 (MMP-2; a) and tissue inhibitor of metalloproteinase-2 (TIMP-2; b) antibodies (DAB, X200) of infected control liver sections of mice sacrificed at the 10**^**th **^**week post infection showing moderate and mild positively stained hepatocytes and granuloma (fibroblast and inflammatory cells; arrows), respectively.** Sections taken from livers of mice treated with PZQ (500 mg/kg/day, p.o., for 2 consecutive days) showing moderate (a) and mild (b) positively stained hepatocytes, and granuloma cells (arrows). Sections taken from livers of mice treated with telmisartan (10 mg/kg/day, p.o., for 5 weeks) showing moderate **(a)** and mild **(b)** positively stained hepatocytes, and granuloma cells (arrows). Sections taken from livers of mice treated with PZQ combined with telmisartan showing moderate **(a)** and mild **(b)** positively stained hepatocytes and granuloma cells (arrows).

As can be seen in Figure 4, treatment with TELM started at the 10^th^ WPI failed to show any significant change in MMP-2 hepatic expression, as compared to the infected control group. However, it caused a 76.77% reduction in TIMP-2 expression, compared to the infected control group. PZQ treatment gave a similar pattern to that of TELM. Thus, the drug did not significantly affect MMP-2 expression, as compared to the infected control group. Meanwhile, a 46.14% reduction in TIMP-2 expression was observed in the PZQ-treated group, as compared to the infected control group. Treatment with PZQ combined with TELM failed to cause any significant change in MMP-2 expression, compared to the TELM- and PZQ-treated groups, as well as TIMP-2 expression, compared to the TELM-treated group (Table 2).

**Figure 5:**
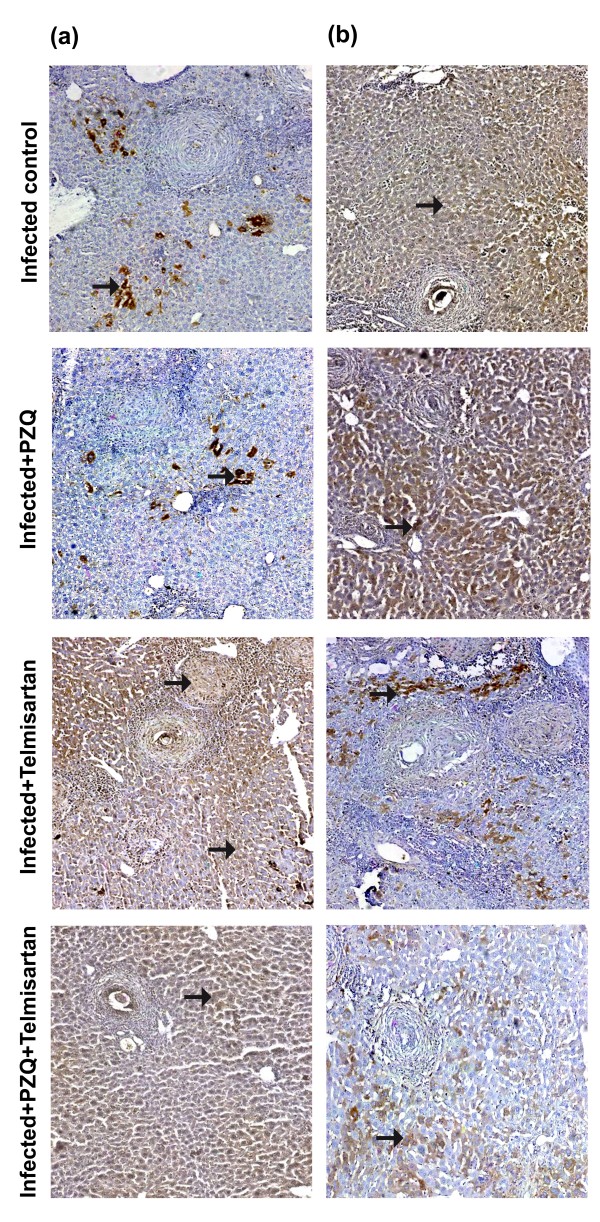
**Immunostain for matrix metalloproteinase-2 (MMP-2; a) and tissue inhibitor of metalloproteinase-2 (TIMP-2; b) antibodies (DAB, X200) of infected control liver sections of mice sacrificed at the 15**^**th **^**week post infection showing mild and moderate positively stained hepatocytes and granuloma (fibroblast and inflammatory cells; arrows), respectively.** Sections taken from livers of mice treated with PZQ (500 mg/kg/day, p.o., for 2 consecutive days) showing mild (a) and moderate (b) positively stained hepatocytes, and granuloma cells (arrows). Sections taken from livers of mice treated with telmisartan (10 mg/kg/day, p.o., for 5 weeks) showing moderate **(a)** and mild **(b)** positively stained hepatocytes, and granuloma cells (arrows). Sections taken from livers of mice treated with PZQ combined with telmisartan showing moderate **(a)** and mild **(b)** positively stained hepatocytes and granuloma cells (arrows).

At the 15^th^ WPI, treatment with TELM showed a 4.25-fold increase in MMP-2 expression and a reduction in TIMP-2 expression by 77.21%, compared to the infected control group. However, PZQ-treated group failed to show any significant change in the expression of either MMP-2 or TIMP-2, as compared to the infected control group. Administration of PZQ in combination with TELM caused no significant change in either MMP-2 or TIMP-2 expression, as compared to TELM-treated group (Table 2; Figure 5).

## Discussion

Hepatic schistosomiasis is considered to be one of the most prevalent forms of chronic liver disease in the world, resulting in morbidity due to complications of liver fibrosis. However, there are few medicines or means available to control and treat fibrosis resulting from schistosomiasis [28]. The immunological response to oviposition in host tissue, especially the liver, which leads eventually to hepatic fibrosis, is considered to be a direct consequence of the schistosomal pathology [29].

Renin angiotensin system blockers have been found to alleviate hepatic fibrogenesis in various experimental models [30-32]. Moreover, there are many studies nowadays suggesting that PPARγ ligands play an important role in inhibiting the progression of liver fibrosis by exerting anti-proliferative and pro-apoptotic actions on activated HSCs, the major collagen producers [16]. The present study has aimed at testing the efficacy of TELM, an AT1 receptor blocker and a PPARγ activator, in both acute and chronic stages of liver fibrosis induced in mice by *S*. *mansoni* infection.

In order to assess the parasitological efficacy of the drugs being tested, both the hepatomesenteric worm load and the oogram pattern were used. Treatment with TELM alone failed to improve any of these parameters in infected mice. This could be attributed to the absence of any toxic effect exerted by TELM on either the eggs trapped in the tissues or the intravascular worms. On the other hand, the reductions in the hepatomesenteric worm load as well as viable egg (mature and immature) percentages observed with PZQ, either alone or combined with TELM, are in agreement with previous studies in which PZQ was used, either alone [33] or in combination with a proposed enhancer such as *Nigella sativa* oil [34], or dietary zinc supplement [35].

Mice that received TELM treatment showed significant reductions in the mean granuloma diameters as well as the estimated fibrotic areas in both sets. These results are in accordance with the results obtained by El-Lakkany *et al*. [36] who showed that losartan, an AT1 receptor blocker, resulted in some healing of the granulomatous hepatic lesions in *S*. *mansoni*-infected mice as could be observed from a reduction in the mean granuloma diameter. The authors suggested an anti-inflammatory pathway through which the drug might have suppressed the immune-mediated reaction to oviposition. It was also found that inhibition of Ang II produced changes in the mechanisms of oxidative stress, especially at the mitochondrial level, indicating an anti-inflammatory effect [37]. Furthermore, the *in vitro* finding that AT1 receptors are expressed on activated human HSCs and are, thus, likely to be increased in number as stellate cells proliferate, and that the binding of Ang II to these receptors induces contraction and proliferation of these cells [14], provides evidence for a potential pathway through which AT1 receptor blockers might alleviate hepatic fibrosis. In addition, Hazra *et al*. [15] reported that PPARγ depletion is associated with HSC activation, whereas HSC quiescence is induced by increasing PPARγ expression, which has been found to inhibit activation markers such as α-smooth muscle actin and collagen. Accordingly, we can conclude that the reduction in the mean granuloma diameter and the fibrotic area, as measures of the degree of fibrosis, caused by TELM treatment, could be attributed to the anti-inflammatory activity as well as its inhibitory effects on HSCs activation, being an AT1 receptor blocker and a PPARγ activator.

The reduction in the granuloma size, following PZQ treatment, has been previously reported by Botros *et al*. [38]. The authors noted a reduction in T-helper cells in the bilharzial granuloma and stated that the granuloma size reduction after PZQ treatment could be the result of the inhibition of the inflammatory mediators released locally at the site of the granulomatous inflammation.

Collagen turnover and ECM remodeling that occur during various physiological and pathological processes including tissue repair, wound healing, fibrosis, and tumor invasion are largely dependent on the regulation of MMP and TIMP activities [39]. In the current study, liver sections of *S*. *mansoni*-infected mice belonging to both sets, showed moderate expressions of both MMP-2 and TIMP-2. This is because the expression of both genes is up-regulated and mainly expressed by HSCs during fibrogenesis. However, during liver fibrosis resolution, as TIMP expression declines, the persistence of MMP-2 may permit collagen degradation [40]. It was found that MMP-2 expression increased and remained elevated during experimental fibrogenesis induced by carbon tetrachloride (CCl_4_) [41]. Yet, high levels of TIMP-2 in the fibrotic liver were capable of inhibiting active MMP-2 [42].

TELM alone increased MMP-2 expression in the second set, and decreased TIMP-2 expression significantly in both sets. The study of Cheng *et al*. [39] showed that activating PPARγ by curcumin treatment increased MMP-2 activity significantly and that this effect was weakened by the specific PPARγ antagonist. In addition, Yu *et al*. [16] demonstrated that PPARγ activation significantly reduced expression levels of TIMP-2 in liver tissue suggesting that it has dual inhibitory effects on HSC activation through inhibiting collagen production and stimulating matrix degradation. Thus, it can be concluded that the observed elevation in MMP-2 and the decline in TIMP-2 expression may have contributed to collagen degradation and a consequent decrease in granuloma diameters and fibrotic areas following TELM treatment.

PZQ treatment alone did not show significant elevation in MMP-2 expression compared to infected controls of both sets. Meanwhile, it caused a reduction in TIMP-2 expression at the 15^th^ WPI while no change was observed at the 10^th^ WPI. These results are in agreement with the study of Singh *et al*. [43], who showed that PZQ treatment did not cause significant changes in MMP-2 expression in *S*. *mansoni*-infected mice, whereas TIMP-2 expression showed little or no change.

Previous studies have shown that in the evolution of the granulomatous response to the *S*. *mansoni* eggs, TGF-β1 is produced and may modulate inflammation and hence regulate fibrogenesis [43,44]. In the current study, TELM caused significant reductions in serum TGF-β1 levels in both sets. A relevant study [32] showed that olmesartan; an AT1 receptor antagonist, succeeded in reducing plasma levels of TGF- β1 in bile duct-ligated rats. Bataller *et al*. [12] also showed that Ang II is a potent inducer of TGF-β1 production in cultured HSCs and *in vivo*. The previously mentioned results suggest that there is a close relationship between Ang II and TGF-β1. Accordingly, the current study reinforces the prevailing hypothesis that TGF-β1 in fibrotic disease depends, at least in part, on Ang II generation. Consistent with the previous results, also is the study of Chen *et al*. [28] who demonstrated that PPARγ ligand, rosiglitazone, can reduce inflammation and liver fibrosis with *Schistosoma* infection by reducing TGF-β1 expression.

Results of the current work revealed that the reductions in serum TGF-β1 levels observed with PZQ were significantly higher than those observed with TELM. This could be attributed to the anti-helminthic activity of PZQ, which leads to the death of viable eggs and prevents their further deposition, which is considered to be the triggering stimulus for the multiple signalling pathways involved in the generation of TGF-β1 in this model. Telmisartan, however, affects only two of these pathways i.e. Ang II and PPARγ. The reduction in TGF-β1 levels elicited by PZQ is consistent with the studies of Singh *et al*. [43]; El-Lakkany & Nosseir [44], and El-Lakkany *et al*. [45] that revealed a marked drop (amounting to 70% in the first study) in the TGF-β1 expression caused by PZQ in *S*. *mansoni*-infected mice.

In the current study, combined TELM and PZQ treatment generally failed to show significantly better results from using either TELM or PZQ alone, provided that the former is given at a dose of 10 mg/kg/day for 5 weeks and the latter is given early in a full curative dose (500 mg/kg/day for 2 consecutive days) as previously mentioned. Hence, this combination did not seem to have an added benefit. This could be attributed to the reduction in egg deposition caused by early PZQ treatment, which is the injurious triggering stimulus for the activation of HSCs. Consequently, this leads to the reduction in the expression of AT1 receptors available for TELM blockade as well as less reduction in PPARγ receptor expression. Thus, in a combined regimen, early treatment with PZQ may have reduced the anti-fibrotic effects observed when compared to using TELM alone. However, a recent study [46] demonstrated an anti-fibrotic effect of PZQ (300 mg/kg, given twice daily for 30 days) at the 8^th^ and 15^th^ WPI in mice with advanced chronic schistosomiasis as well as in CCl_4_-induced liver fibrosis. Yet, further studies examining the efficacy of combining TELM to PZQ, which may require using different doses of both drugs, are recommended to support this speculation.

## Conclusion

In conclusion, our study showed a significant reduction of liver fibrosis following treatment with TELM, an AT1 receptor blocker and a PPARγ ligand, in a murine model of hepatic fibrosis induced by *S*. *mansoni* infection. The anti-fibrotic effect of this compound could be due to down-regulation of TGF-β1 as well as increasing the hepatic expression of MMP-2 relative to TIMP-2. Thus, its potential improvement of fibrotic markers makes the drug a particularly strong candidate for treating hepatic fibrosis. Moreover, TELM may be more efficient than other AT1 receptor blockers such as losartan or candesartan, in suppressing liver fibrosis, due to activation of PPARγ receptors besides AT1 receptor antagonism.

The RAS and the PPARγ receptors are important targets for the treatment of fibrosis. Searching for agents that can affect multiple signalling pathways involved in hepatic fibrosis is recommended. Additionally, testing the efficacy as well as the safety of prolonged PZQ treatment as an anti-fibrotic, independent of its anti-helminthic activity, particularly in the late stages, is also recommended. Finally, the effectiveness of TELM as a choice for patients treated for hypertension or heart failure associated with liver affection needs further research.

## Competing interests

All authors declare that they have no competing interests.

## Authors’ contributions

ASE and EFA conceived and designed the experiments; YMA performed the experiments and the biochemical analysis; OAH performed the histopathological, morphometric, and immunohistochemical studies; SSM performed the parasitological studies; ASE, OAH, EFA, and YMA analyzed the data; ASE, OAH, EFA, and YMA prepared the manuscript. All authors have read and approved the final version of the manuscript.
